# Correlates of premature pap test screening, under 25 years old: analysis of data from the CONSTANCES cohort study

**DOI:** 10.1186/s12889-021-10603-4

**Published:** 2021-03-25

**Authors:** Stéphanie Mignot, Virginie Ringa, Solène Vigoureux, Marie Zins, Henri Panjo, Pierre-Jean Saulnier, Xavier Fritel

**Affiliations:** 1 Department of General Practice, University of Poitiers, France: 3 rue de la Milétrie, 86000 Poitiers, France; 2 CESP Centre for research in Epidemiology and Population Health, U1018, Gender, Sexuality and Health team, University Paris-Saclay, University Paris-Sud, UVSQ, Villejuif, Ined France; 3 Obstetrics & Gynecology department, Hôpital Bicêtre, GHU Sud, AP-HP, Faculty of Medicine, Univ of Paris Sud, F-94276 Le Kremlin Bicêtre, France; 4 Epidemiological Population-Based Cohorts Unit, INSERM UMS 11,Villejuif, France, University of Paris-Descartes, Paris, France; 5 Clinical Investigation Centre CIC1402 INSERM, School of Medicine, Poitiers University, CHU Poitiers (University Hospital), 86000 Poitiers, France; 6 Clinical Research Centree CIC1402, INSERM, Department of Obstetrics, Gynecology, and Reproductive Medicine Poitiers University Hospital Centre, University of Poitiers, Poitiers, France

**Keywords:** Contraception, Pap test, Overscreening

## Abstract

**Background:**

Many countries currently recommend that screening for cervical cancer begin at the age of 25 years. Premature screening (before that age) could lead to unnecessary follow-up examinations and procedures that turn out to be useless. Our objective is to ascertain if the use of particular contraceptive methods are associated with premature screening.

**Methods:**

This cross-sectional study based on the CONSTANCES cohort enabled us to include 4297 women younger than 25 years. The factors associated with premature screening were modeled by logistic regression. Missing data were handled by multiple imputations. The multivariate analyses were adjusted for sex life, social and demographic characteristics, and health status.

**Results:**

Nearly half (48.5%) the women younger than 25 years had already undergone premature screening. Women not using contraceptives (aOR 0.3, 95% CI 0.3–0.5) and those using nonmedicalized contraceptives (condom, spermicide, etc.) (aOR 0.5, 95% CI 0.4–0.6) had premature screening less often than women using birth control pills. Higher risks of premature screening were observed in 20-year-old women (aOR 2.7, 95% CI 2.2–3.3) and in those with more than 5 lifetime partners (aOR 2.5, 95% CI 2.0–3.1), compared respectively with women who were younger and those with 5 or fewer lifetime partners.

**Conclusion:**

Young women using contraceptives that require a doctor’s prescription are exposed to premature screening more often than those not using contraception and those with nonmedicalized contraceptives.

## Background

The *human papillomavirus* (HPV) is responsible for nearly all cervical cancers. It is the most common of the sexually transmissible virus infections, with especially high prevalence rates of 29 to 45% observed in European women aged 20–24 [1]. In most cases, viral clearance enables spontaneous recovery; when cervical dysplasia is identified in a young woman, the probability of regression is 91% at 36 months [2]. The cancer occurs many years after the contamination in the absence of viral clearance. The mean age at diagnosis of this cancer in Europe is 51 years [3]. A vaccine against HPV exists and was approved for use more than a decade ago in France. It is intended for young girls aged 11 to 14 years, with catch-up until 19 years. Cervical cancer screening by Pap smears is nonetheless still recommended for women who have been vaccinated in France [4]. Different studies have shown the value of the Pap test in reducing the incidence of cervical cancer [5]. Many countries now recommend that cervical cancer screening (CCS) with this test start at the age of 25 years (Belgium, Italy, Ireland, Greece, the UK, and France) [6]. This de-intensification of CCS has the potential to reduce overdiagnosis and overtreatment of cervical abnormalities and the additional harms associated with them. This is particularly relevant for women under 25 years, among whom cervical cancer incidence and mortality are extremely low [7]. However, premature screening exposes women to overscreening of lesions likely to disappear spontaneously in most cases, and accompanied by unnecessary follow-up examinations (repeated Pap smears, colposcopy, and biopsies), and additional procedures (conizations, laser treatment, and cryotherapy) [8, 9]. Another consequence of overscreening is that young women will develop a fear of gynecologic examinations that will cause them to not seek medical care to obtain contraception [10]. It is likely that more harm than good may be caused by a premature pap test.

Few studies have focused on the factors influencing premature CCS (before the age of 25 years) [11]. Consultations for contraception can be the occasion for a Pap smear earlier than required by age, even though it is no longer recommended. Women who use contraceptives that require a consultation with a physician and a prescription, such as an intrauterine device (IUD), pills, microinsert, etc. (referred to hereafter as medical contraceptives) may have premature Pap smears more often than women using contraceptives that do not require prescription (condoms, withdrawal, natural spermicides, the rhythm or Knaus-Ogino methods^1^ etc., all referred to here as nonmedical contraceptives) or who use none at all. That is, among women aged 25 years or older, those who use an IUD are more up to date on Pap smears than those using nonmedical or no contraceptives [12], precisely because a visit to a physician and in particular a gynecologist to obtain contraception is an opportunity for a Pap smear.

The objective of this analysis is to examine if an association exists between the types of contraception used by young women and premature screening by Pap tests, while taking into account other factors, such as social and demographic characteristics, sexual health, sexual orientation, and health status.

## Methods

### Population study

This cross-sectional study is based on the CONSTANCES cohort of volunteers aged 18–69 years in 22 selected health screening centers from the principal regions of France (www.constances.fr). Participants are randomly selected from adults covered by the National Health Insurance Fund (i.e., salaried workers, currently working or retired, and their family members), stratified by age, sex, region, and socioeconomic position. The data considered here were collected at inclusion and come from the questionnaires about lifestyle, women’s health, and occupational exposures. The data were collected from 2012 to 2019. This analysis covers the women aged younger than 25 years recruited between January 2012 and April 2019. It excluded the following women: those who a priori did not require contraception, that is, who reported a hysterectomy or bilateral oophorectomy; those who reported they have never had sex; and those who reported that they have had cervical cancer.

. Body mass index (BMI) was obtained from participants’ weight and height measurements, collected at the initial medical examination.

### CONSTANCES cohort

CONSTANCES collects data on personal, environmental, behavioral, occupational, and social factors from questionnaires self-administered at inclusion and annually thereafter, mailed to and returned by participants (or collected in the health centers). This general-purpose epidemiological cohort is designed to study a wide range of health problems in various disciplines in the general population. Its long-term objective is to follow 200,000 members (men and women) of the French population, aged 18 to 69 years; inclusion in this cohort began in 2012 [13]. After enrolment, participants are followed up by an annual self-administered questionnaire sent to their homes (paper or web-based), and they are invited for a new health examination every 5 years.

### Outcome measurements

The variable to be explained was premature screening (having had a Pap smear before the age of 25 years). Women younger than 25 years who had answered “Yes” to the question “Have you ever had one or more Pap tests (smears taken from the cervix)?” were considered to have undergone premature screening.

### Contraception and sex life

For contraception, the principal explanatory variable, we distinguished the different types of contraceptives according to their degree of medicalization *(does or does not require prescription and follow-up by a healthcare professional)* and then in more detail, into 5 contraceptive choices:

- Medical contraceptives: Combined estrogen-progestin or progestin-only contraceptives, regardless of their form (oral, patch, ring, injection), which require at least one medical consultation annually for their prescription. Because this contraceptive was the most frequently used, it was the reference category. Contraception by IUD: a device must be placed by a physician (or midwife) and changed every 3 to 10 years. Women who use it are advised to have annual clinical examinations. Contraception by implants, which must be placed by a physician (or midwife) and changed every 3 years, but for which annual consultations are not required (only a check-up visit 3 months after its placement is recommended).
- Nonmedicalized contraception: These are the contraceptives that require no medical visit (condom, withdrawal, spermicides, and rhythm or Knaus-Ogino methods).
- Absence of contraception: This category included the women who reported sexual relations but not contraceptive use, although they did not want to become pregnant.

Women answered the following questions about their sex lives and reproduction: sex of partners (male/female/both/do not wish to answer DNWA), number of lifetime partners (number/DNWA), new partner in the past 12 months (yes/no/DNWA), pain during intercourse (never/sometimes /often/always), sexual satisfaction (currently your sex life seems: not at all satisfactory/not very satisfactory/satisfactory/very satisfactory /DNWA/not applicable). The responses to this question were summarized as satisfactory and unsatisfactory, with women who answered “very satisfactory or satisfactory” classified as satisfied. The women considered to have pain during intercourse (dyspareunia) were those who answered “often” or “always”.

### Social and demographic characteristics

The other indicators considered were age, parity, civil status, and geographic origin defined according to place of birth, financial difficulties, forgogone medical care. Educational level was defined by the highest diploma completed: less than the baccalaureate or school-leaving exam (“bac”), passed the “bac”, some post-secondary education, other diplomas.

### Health status

A specific question allowed respondents to classify their health status as good, medium, or poor.

The categories for smoking were: current smoker, ex-smoker, non-smoker; for alcohol consumption: irregular consumption (less than 4 times a month), regular consumption (one to several times a week), and not currently; for marijuana use the question was “have you ever used marijuana in your lifetime?” The possible responses were yes/no/DNWA. The weight and height of each participant were measured at the medical examination at the health center and enabled calculation of her body mass index (BMI). This variable was introduced in categories according to the WHO classification (< 18.5 underweight, 18.5–24.9 normal weight, 25.0–29.9 overweight, 30.0–39.9 obese, and > 40 morbidly obese).

### Statistical analyses

Quantitative variables (age and BMI) were described by their means and standard deviations, and the qualitative variables as percentages. The quantitative variables were then discretized into categories. To assess the association between premature CCS and the categorical variables, we performed Chi2 or Fisher’s exact tests of independence.

To understand the role of each variable, we first studied the associations between the explanatory variables and premature CCS in a univariate analysis. Variables were retained when they were associated with premature CCS with a *P* value < 0.05. They were then included in 3 separate thematic logistic regressions (contraception and sex life, social and demographic characteristics, and health status). These models were simplified by backward elimination. A final model including the associated variables for each thematic model (at *P* <  0.05) also underwent the backward elimination procedure. The associations between premature CCS and the variables of interest were expressed by adjusted odds ratios, and their 95% confidence intervals.

Missing data were handled by using multiple imputations with fully conditional specification (SAS 2013) and assuming missingness at random (MAR). To make the MAR assumption more plausible, every previously described variable was used for the imputation model [14, 15], including the outcome. Excluding the outcome from the imputation model could have hidden some associations, and including it did not change the standard deviations [16]. Ten complete datasets were created. This method, known as MID (multiple imputations, then deletion), uses information about the dependent variable in the imputation model (as well as the standard imputation method), but cases with imputed outcomes are deleted before analyses [17]. Overall, 10% or more of the data were missing from the following variables: satisfactoriness of sex life (13.06%), number of lifetime partners (44.2%), socio-professional categories (14.64%), and type of professional who performed the Pap test (52.04%). The missing data rate was 4.6% for the Pap test status.

The analyses were performed with SAS software, version 9.3.

EthicsThe national council on statistical information (CNIL) approved the CONSTANCES study (CNIL authorization n°910,486). An additional related application to the CNIL was approved on January 25, 2016 (CNIL authorization n°1,881,675).

## Results

**Fig. 1:**
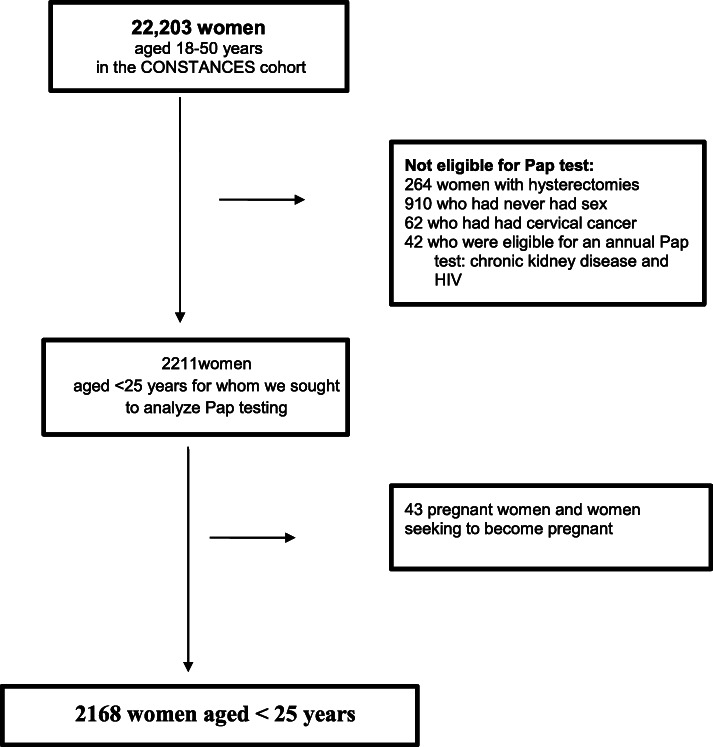
selection for analyzed sample

Population sampleIn all, 4297 women aged 18–24 years answered the CONSTANCES questionnaires for our analysis (Fig. 1). The women younger than 20 years accounted for 14.4% (620) of the sample. Overall, 48.5% (2084) of those younger than 25 years had already had at least one Pap test. Gynecologist had performed 41.9% of the tests, general practitioners 4.61%, and other physicians 1.44%. The question about the Pap test remained unanswered by 198 women in our sample.

The univariate analysis showed that the category of contraception used was associated with having had Pap tests: the women using no or nonmedicalized contraception (OR 0.4, 95% CI 0.3–0.5; OR 0.6, 95% CI 0.5–0.7) had a lower risk of premature screening than those who used birth control pills. The women who had an IUD were at the highest risk of having already undergone a CCS (OR 2.7, 95% CI 2.1–3.6). Those who had had more than 5 lifetime sexual partners also had a higher risk of having had a premature Pap test (Table 1). Among the variables concerning their sex lives, neither pain during sexual intercourse nor a new partner during the past 12 months was associated with premature screening.

**Table 1:** Characteristics associated with premature Pap test screening; multivariate analysis: women aged under 25 years, contraception and sex life

Contraception and sex life				
	OR [95% CI]	***P***	aOR [95% CI]	***P***
**Contraceptive practices**				
Short duration (hormonal)	Réf,	*–*	Réf,	**–**
Long duration (implant)	0.9 [0.5–1.7]	*0.78*	0.8 [0.4–1.7]	0.60
Long duration (IUD)	2.7 [2.1–3.6]	*< 0.0001*	2.1 [1.5–2.8]	< 0.0001
Nonmedical (condom, natural, etc)	0.6 [0.5–0.7]	*< 0.0001*	0.5 [0.4–0.6]	< 0.0001
No contraception	0.4 [0.3–0.5]	*< 0.0001*	0.3 [0.3–0.5]	< 0.0001
**Sexual orientation**				
Heterosexual	Réf,	*–*	Réf,	*–*
Lesbian	0.4 [0.3–0.7]	*0.0003*	0.5 [0.3–0.9]	*0.0069*
**Sex life satisfactory**				
Yes	Réf,	*–*	*–*	*–*
No	0.9 [0.8–1.1]	*0.37*	*–*	*–*
**Pain during intercourse/dyspareunia**				
Often	1.1 [0.9–1.4]	*0.36*	*–*	*–*
Never	Réf,	*–*	*–*	*–*
**New partner in the past 12 months**				
Yes	0.9 [0.8–1.0]	*0.13*	*–*	*–*
No	Réf,	*–*	*–*	*–*
**Number of lifetime partners**				
Fewer than 6	Réf,	*–*	Réf,	*–*
6 to 29	2.8 [2.3–3.4]	*< 0.0001*	2.5 [2.0–3.1]	*< 0.0001*
30 to 50	3.6 [0.4–30.1]	*0.22*	5.1 [0.5–54.8]	*0.15*

The women at the highest risk of premature Pap test screening were either older than 20 years or had already had a child. Health status and financial difficulties did not influence the risk of a premature Pap test for CCS (Table 2).

**Table 2:** Characteristics associated with premature Pap test screening; multivariate analysis: women aged under 25 years, socioeconomic and demographic characteristics

Socioeconomic and demographic characteristics				
	OR [95% CI]	***P***	aOR [95% CI]	***P***
**Age groups**				
18–19 years	Réf,	*–*	Réf,	*–*
20–24 years	2.7 [2.2–3.3]	*< 0.0001*	2.7 [2.2–3.3]	*< 0.0001*
**Parity**				
Nulliparous	Réf,	*–*	Réf,	*–*
Primiparous	2.5 [1.6–3.9]	*< 0.0001*	2.1 [1.2–3.5]	*0.0088*
**Educational level**				
Below Baccalaureate	0.6 [0.5–0.8]	*< 0.0001*	0.8 [0.6–1.0]	*0.09*
Baccalaureate	0.5 [0.4–0.5]	*< 0.0001*	0.6 [0.5–0.7]	*< 0.0001*
Post-secondary school	Réf,	*–*	Réf,	*–*
Other diplomas	0.7 [0.4–1.3]	*0.27*	0.6 [0.3–1.3]	*0.19*
**Civil status**				
Maried or civil union	Réf,	*–*	Réf,	*–*
Single	0.5 [0.4–0.7]	*< 0.0001*	0.6 [0.4–0.8]	*0.0025*
Separated, divorced, widowed	0.8 [0.4–1.5]	*0.29*	0.5 [0.2–1.4]	*0.18*
**Geographic origin**				
metropolitan France	Réf,	*–*	–	–
French overseas departments and territories	0.8 [0.5–1.3]	*0.46*	–	–
Europe	1.1 [0.7–1.7]	*0.59*	–	–
Africa/Asia	0.7 [0.5–1.1]	*0.13*	–	–
Other	1.6 [0.8–3.2]	*0.19*	–	–
**Has forgone medical care**				
No	Réf,	*–*	Réf,	*–*
Yes	1.4 [1.2–1.6]	*0.0002*	1.8 [1.1–2.9]	*0.024*
**Financial difficulties**				
In the past	1.4 [1.1–1.7]	*0.001*	1.0 [0.7–1.3]	*0.81*
Currently	1.3 [1.1–1.6]	*0.009*	0.8 [0.6–1.2]	*0.27*
For a long time	1.7 [1.2–2.3]	*0.001*	1.0 [0.6–1.5]	*0.87*
Never	Réf,	–	Réf,	–

In the multivariate analysis, the use of a contraceptive such as the IUD remained associated with the risk of a premature Pap test, compared with women who used short-term hormonal contraception (such as the pill or patches) (aOR 2.1, 95% CI 1.5–2.8); the latter in turn were at higher risk than those who used no or nonmedical contraception (aOR 0.5, 95% CI 0.4–0.6) (Table 1). Young women who reported they were lesbians also had less risk of a Pap smear than self-reported heterosexuals. Finally, risk was higher among those who reported more than 5 lifetime sex partners than among those with fewer partners.

Socioeconomic and demographic characteristics were still associated with overscreening: The women aged 20 years or older were at higher risk of a premature Pap test than those younger than 20 years (aOR 2.7, 95% CI 2.2–3.3) (Table 2). The married women and those who had already had a pregnancy were at higher risk of premature Pap testing than women those who were, respectively, single and nulliparous. Having forgone medical care for financial reasons remained associated with the risk of a premature Pap test (aOR 1.8, 95% CI 1.1–2.9). Health status did not influence overscreening (Table 3).

**Table 3:** Characteristics associated with premature Pap test screening; multivariate analysis: women aged under 25 years, health status

Health status				
	OR [95% CI]	***P***	aOR [95% CI]	***P***
**Perceived health status**				
Good	Réf,	*–*	–	–
Medium	0.9 [0.7–1.1]	*0.27*	–	–
Poor	1.4 [1.0–2.1]	*0.086*	–	–
**Body mass index**				–
BMI < 18 malnourished/underweight	0.7 [0.6–0.8]	*< 0.0001*	0.8 [0.5–1.1]	*0.16*
18 < =BMI < 25 normal	Réf,	*–*	Réf,	*–*
25 < =BMI < 30 overweight	1.0 [0.8–1.2]	*0.93*	1.1 [0.8–1.6]	*0.64*
30 < = BMI < 40 obese	0.9 [0.7–1.2]	*0.34*	0.4 [0.1–1.3]	*0.12*
**Smoking status**				
Smoker	1.3 [1.1–1.5]	*0.0002*	1.1 [0.9–1.2]	*0.77*
Non-smoker	Réf,	*–*	Réf,	*–*
**Alcohol consumption**				
Drinks alcohol regularly	1.5 [1.3–1.7]	*0.0001*	1.2 [1.0–1.4]	*0.88*
Drinks alcohol irregularly	Réf,	*–*	Réf,	*–*
Does not drink alcohol	1.1 [0.9–1.4]	*0.36*	1.1 [0.8–1.5]	*0.47*
**Marijuana use**				
Yes	1.3 [1.2–1.5]	*0.0001*	1.0 [0.9–1.2]	*0.87*
No	Réf,	–	Réf,	*–*

## Discussion

Our analysis from the CONSTANCES cohort allowed us to show that a high proportion of the women younger than 25 years had had a premature Pap test, a finding indicating that their clinicians are not adhering to guidelines. Those with an IUD had the greatest risk of CCS before it was necessary, useful, or recommended. The women who used the pill were at a higher risk than those who used either no or nonmedicalized contraception. The women with a child and those with more than 5 lifetime sexual partners had also had a premature Pap test more often.

### Limitations in the database

The CONSTANCES study population had several advantages as a cohort for our study: a diverse population, a very large sample size, a large number of diverse questions asked of women that enabled us to explore the association between the rate of Pap tests performed in violation of guidelines, and factors such as BMI, financial difficulties, sexual function, and their effects. The survey did not ask for information about these women’s religious or cultural practices, which are nonetheless factors that might play a role in the performance of CCS [18]. Information about age at first sexual relations would have been interesting because it could influence the performance of a premature Pap test. Among the women in the sample, only 11 reported having had salpingitis; accordingly, we could not use this variable in the analysis. The study unfortunately did not collect any data about vaccination against HPV, which would clearly have been interesting. In 2018, the rate of vaccination coverage in France was 29% for young girls for a single dose and 24% for a complete course of anti-HPV vaccine [4]. Since the Pap test remains recommended, despite the vaccination, its effect on the performance of the Pap test may be modest, especially in view of the exceedingly low French vaccination coverage.

Women with postsecondary education were overrepresented in the sample: 47.2% of the women had some higher education, compared with 42% in the general population of French women older than 15 years (data about educational level are not presented) [19]. Moreover a possible nondifferential classification bias exists: some women might have reported that they had had a Pap test although they might only have had a speculum inserted for a gynecologic examination or a simple sample taken to test for an infection. It is possible that not all of the women were well informed about the performance, objective, and limitations of the Pap test [20].

The women who had premature Pap test screening used contraception that required a prescription more often than women who had not had this test. Langille and Rigby similarly concluded that adolescents using the pill have a higher risk of having already had a Pap test than those who used no contraceptives or had used a condom the last time they had sexual intercourse [21]. The fact of seeing a physician can promote CCS. The social position of women who consult gynecologists is fairly high; women in lower socioeconomic categories see general practitioners more often, and these physicians perform Pap tests less often [22]. The women who adhere most closely to screening tests often come from higher socioeconomic levels [23]. They are also the women who use the pill or an IUD most often for contraception [24]. The concept of prevention may be socially embedded in their lifestyle. They may request Pap tests more often than women with lower educational levels. A study exploring changes in CCS after the 2009 changes in the American College of Obstetricians and Gynecologists’ guideline showed that overscreening increased significantly in women younger than 30 years [25]. We found that women with a child, compared to nulliparous women, and those who were married, compared to those who were single, were at higher risk of premature Pap tests. Studies have found that marital status has an influence on women’s health [26].

It is difficult to know the proportion of overscreening that is due to women’s requests relative to that associated with the healthcare professionals’ lack of adherence to guidelines. This overscreening is undoubtedly due to both physicians and their patients. Women often ask for Pap tests. In a study published in 2005 in the United States, 69% of the women, independent of socioeconomic level, reported they wanted to have annual Pap tests, even if their physician recommended they do so only every 3 years. Half the women thought that the new guidelines were intended to reduce costs, rather than to improve medical care [27]. Moreover the information campaigns promoting screening hide the disadvantages and risks of premature Pap tests. Although it can be difficult to convince women to adhere to screening, it can also be difficult to convince them that screening is not necessary. Our study shows that it is necessary to make young women and physicians understand that inappropriate prevention has real harms: both women and physicians find it difficult to recognize that screening examinations are not risk-free. Young women especially are best served by avoiding CCS until they turn 25.

Our results suggest that social characteristics, sexual behaviors, and contraception use may all contribute to overscreening and that both doctors and patients probably play a role in it. Moreover, more attention by researchers to the harms of excessive screening is needed. Our study could help clinicians to understand the kinds of situations that can lead to overscreening, so that they then contribute to modifying women’s medical attitudes.

Persons under 25 years use social media frequently. This technology influences human behavior in part by improving information sharing. New medical practices, including changes in or elimination of old practices, could be explained to the public on social media. Medical authorities might also use these contemporary means of communication to amplify medical messages toward specific populations [28].

In the USA, physicians tend not to adhere to the guidelines about the ages for starting and ending CCS [29]. They report that the guidelines strongly influence their practices, but 95% of them would perform Pap tests for sexually active 18-year-olds who have come for their first gynecologic visit. Half of them would perform it even for 18-year-olds who are not sexually active. US general practitioners (GPs) tend to follow the guidelines for screening ages (lifetime starting and stopping CCS) more than gynecologists-obstetricians do [29]. In France, GPs can find it difficult to follow some guidelines when they prioritize the doctor-patient relationship: if a young woman wants a Pap test, they will perform it, even knowing that it is not recommended. GPs adjust their practices to their patients’ individual characteristics (worries, beliefs etc.), which may explain why they do not always adhere to clinical practice guidelines [30].

## Conclusion

Campaigns to teach best practices to doctors by means other than guidelines appear necessary. For women younger than 25 years, the prescription of contraceptives must be dissociated from the practice of a routine gynecologic examination or a Pap test. Among this group, this will result, independently of the cost savings involved, in reducing the number of unnecessary and mutilating interventions (conization). Physicians perhaps lack adequate knowledge of the consequences of premature screening. Our data could be useful for targeting awareness campaigns aimed at Doctors and women about the need not to have CCS too early.

Our study suggests that the use of specific types of contraception is associated with the performance of Pap tests: young women who use nonmedical contraceptives have fewer Pap tests, and those using an IUD have the most. Application of the recommendation about the need to avoid Pap tests for women younger than 25 years appears inadequate. Young women see clinicians for contraception. That should be provided, along with necessary information but not unnecessary and potentially harmful procedures.

## Data Availability

Data and material are available from stephanie.mignot@univ-poitiers.fr

## Abbreviations

BMI: Body mass index
CCS: Cervical cancer screening
CNIL: National council on computer privacy Ou data protection
GP: General practitioner
HPV: *Human papillomavirus*
IUD: Intrauterine device
MAR: Missingness at random
MID: Multiple imputation then deletion
UK: United Kingdom
WHO: World Health Organization

## Footnotes

1 This methods is a so-called “natural” calendar-based contraceptive method, better known as the rhythm method, which involves avoiding sexual intercourse during the period when the woman can become pregnant.
